# Development and Temporal Validation of a Discharge-Based Administrative Data Risk Model for 30-Day Unplanned Readmission After Endometrial Cancer Staging Surgery

**DOI:** 10.3390/healthcare14152249

**Published:** 2026-07-23

**Authors:** Lingdan Lu, Jiong Ma, Yanyan Ma, Yue Pang, Pu Cheng

**Affiliations:** 1 Department of Nursing, The Second Affiliated Hospital Zhejiang University School of Medicine, Hangzhou 310009, China; 2 Department of Gynecology, The Second Affiliated Hospital Zhejiang University School of Medicine, Hangzhou 310009, China; majiong@zju.edu.cn (J.M.); myy0908@zju.edu.cn (Y.M.); 3 Department of Anesthesia Surgery, The Second Affiliated Hospital Zhejiang University School of Medicine, Hangzhou 310009, China; 2613017@zju.edu.cn

**Keywords:** endometrial cancer, unplanned readmission, risk prediction model, temporal validation, postoperative outcomes

## Abstract

**Background**: Postoperative complications after staging surgery for endometrial cancer often occur after discharge, and 30-day unplanned readmission is an important post-discharge outcome. Existing models lack same-day usability at discharge and sufficient validation, limiting discharge-time risk stratification and transitional care planning. This study aimed to develop and validate a discharge-time risk prediction model based on administrative data for 30-day unplanned hospital readmission among patients undergoing staging surgery for endometrial cancer. **Methods**: This retrospective cohort study was conducted in accordance with the TRIPOD reporting guidelines. We constructed a nationally representative adult cohort using the Healthcare Cost and Utilization Project Nationwide Readmissions Database (HCUP-NRD) from 2016 to 2022, including index hospitalizations discharged between January and November to ensure complete 30-day follow-up. Variables available at discharge (demographics, payer/income, presentation, inpatient course, discharge disposition, hospital characteristics, Elixhauser index) were included; age and length of stay were modeled with restricted cubic splines. Complex survey-weighted multivariable logistic regression was used for model development with 1000 bootstrap internal validations. Temporal validation within the NRD was performed using 2020–2021 and 2022 data. Decision curve analysis, subgroup, and sensitivity analyses were conducted. **Results**: A total of 89,627 hospitalizations were included; development and validation cohorts had balanced baselines. Internal validation showed moderate discrimination (AUC 0.719) and good calibration (Brier 0.045; intercept 0.014; slope 0.955). Discharge to home health or institutional care, non-elective or emergency admission, and a higher Elixhauser readmission index (a measure of comorbidity burden) were associated with increased risk. Age and length of stay showed nonlinear associations. Temporal validation yielded a similar performance (AUC 0.708–0.713; Brier 0.043–0.046), and decision curves indicated positive net clinical benefit. **Conclusions**: The model demonstrated stable discrimination and maintained good calibration after temporal validation and recalibration. It represents a candidate discharge-time decision-support tool, based on administrative data, for identifying patients at higher risk of 30-day unplanned readmission, and may support nurse-led transitional care planning. External validation and local recalibration are required before application in other healthcare settings.

## 1. Introduction

Endometrial cancer is a common gynecologic malignancy, and staging surgery remains the core pathway for initial treatment [1]. Advances in minimally invasive surgery and enhanced recovery pathways have shortened hospital stays, making early postoperative complications increasingly likely to occur after discharge and highlighting 30-day unplanned readmission as an important quality and patient safety outcome [2,3]. Postoperative infections, thrombosis, and urinary complications frequently occur after discharge, emphasizing the need for effective post-discharge risk identification [4]. Readmission not only increases costs and bed occupancy but also interrupts subsequent treatment arrangements and affects patients’ experience of nursing care [5].

Discharge readiness assessment, symptom education, medication reconciliation, and transitional follow-up are mostly led by nursing teams; risk stratification may help inform nurse-led transitional care planning [6]. Existing studies have mainly investigated readmission incidence and risk factors using single-center or regional cohorts with heterogeneous gynecologic oncology populations, limiting the availability of evidence specific to endometrial cancer staging surgery [7,8]. Furthermore, some prediction models require tumor staging, surgical details, or laboratory variables that are not routinely available at discharge, reducing their applicability as discharge-time decision-support tools [9,10]. The definition of “unplanned” readmission varies substantially across studies; failure to adequately exclude hospitalizations related to planned therapy can cause outcome misclassification [11]. Changes in medical workflows and discharge resource supply before and after the pandemic increase the risk of temporal drift; models lacking temporal validation cannot ensure long-term, stable application, and interpretable models oriented to nursing practice remain scarce.

Using HCUP-NRD data from 2016 to 2022, we constructed an adult inpatient cohort undergoing staging surgery for endometrial cancer. We defined 30-day unplanned readmission using a standardized algorithm, developed a prediction model based on variables available at discharge, including patient characteristics, inpatient course, discharge disposition, and hospital-level factors, and performed temporal validation in pandemic and post-pandemic populations. This study aimed to develop a discharge-time risk prediction model based on administrative data, temporally validate it within the NRD, and evaluate its discrimination, calibration, and clinical utility.

## 2. Materials and Methods

### 2.1. Data Source and Study Design

This study used the Nationwide Readmissions Database (NRD) of the Healthcare Cost and Utilization Project (HCUP), covering discharge records from 2016 to 2022. The NRD is constructed from multiple State Inpatient Databases and provides a weighted national analytic sample for hospital readmissions. It contains a patient linkage number (NRD_VisitLink) and a days-to-event variable (NRD_DaysToEvent) to track multiple hospitalizations for the same patient across hospitals within the same year. Each NRD year is an independent annual sample, and the same patient or the same hospital cannot be tracked across years. The study design was a retrospective cohort and prediction model study. The research report was written in accordance with the Transparent Reporting of a multivariable prediction model for Individual Prognosis or Diagnosis (TRIPOD) guidelines [12] (see Supplementary File S1). Using the index hospitalization corresponding to staging surgery for endometrial cancer as the starting point, we developed a discharge-time risk prediction model based on administrative data for post-discharge 30-day unplanned readmission and conducted temporal validation within the NRD. The NRD uses a post-stratification method based on hospital characteristics (region, urban/rural and teaching status, bed size, and ownership) to generate discharge-level weights (DISCWT). In the analyses, we combined NRD_STRATUM with the hospital cluster identifier HOSP_NRD and applied a complex survey-weighted design to produce national-level descriptive estimates and variances. The inference level was limited to the national level; we did not conduct regional, state, or single-hospital inference. Institutional Board Review approval was exempted for this study because the NRD database contains deidentified patient information [13].

### 2.2. Study Population and Cohort Construction

#### 2.2.1. Timing and Uniqueness of the Index Hospitalization

The study period was limited to discharges from 1 January 2016 to 30 November 2022. To ensure a complete 30-day follow-up window, the index hospitalization included only records discharged in January–November of each year. Each patient (by NRD_VisitLink) was allowed only one eligible index hospitalization for modeling within each year; if the same patient met the conditions multiple times in the same year, the first was used as that year’s index hospitalization. Cross-hospital same-day/next-day acute transfers were merged into a single continuous hospitalization per the NRD “same-day event/transfer consolidation” rule and were not counted as readmissions.

#### 2.2.2. Case Definition of Endometrial Cancer Diagnosis

ICD-10-CM C54.1 (malignant neoplasm of endometrium) was used as the core diagnosis and was allowed to appear in any diagnosis position. To improve specificity, C55 (malignant neoplasm of uterus, part unspecified) was excluded. When necessary, C54.0, C54.2, C54.3, C54.8, and C54.9 were used as supplementary codes for the sensitivity definition. The final code list is presented in Supplementary Table S1.

#### 2.2.3. Operational Definition of “Staging Surgery”

Staging surgery was required to meet both of the following within the same hospitalization: ① hysterectomy (ICD-10-PCS 0UT9*ZZ series, for example, 0UT90ZZ, 0UT94ZZ); ② pelvic or para-aortic lymph node biopsy/dissection (based on mapping ICD-10-PCS to PR CCSR-corresponding categories with positive flags for “female reproductive system surgery: hysterectomy” and “lymphatic system surgery: lymph node biopsy/dissection”). PR CCSR was used to aggregate all ICD-10-PCS into clinically interpretable categories to maintain consistent definitions across multiple years. The specific versions and code lists for coding and category mapping are provided in Supplementary Table S1.

#### 2.2.4. Inclusion and Exclusion Criteria

Inclusion criteria: ① age ≥18 years; ② meeting the diagnosis and surgical definitions in Section 2.2.2 and Section 2.2.3; ③ discharge outcome was alive and left the hospital (see Section 2.3.5). Exclusion criteria: ① in-hospital death during the index hospitalization; ② discharge disposition was transfer to another acute care hospital or unknown; ③ missing or invalid patient linkage information; ④ discharge in December in any year from 2016 to 2022; ⑤ any record violating NRD quality rules (e.g., suspicious high-frequency readmission flags).

### 2.3. Variables and Outcome Definitions

#### 2.3.1. Primary Outcome: Post-Discharge 30-Day Unplanned Readmission

Per HCUP methods, the first readmission occurring on days 1–30 after the index discharge was defined as the outcome event. The time interval was calculated as the difference in NRD_DaysToEvent between two encounters for the same patient, together with the length of stay (LOS) of the index hospitalization, to ensure the interval was counted from the day of discharge; if multiple readmissions occurred within 30 days, only the first was used.

#### 2.3.2. Determination of “Unplanned Readmission”

“Planned” readmissions were excluded using a two-step approach: ① elective admissions were first removed according to the NRD-derived ELECTIVE variable (generated from ATYPE); ② the CMS Planned Readmission Algorithm (Version 4.0 implementation) was applied to identify further and exclude planned care. Under this algorithm, transplant surgery, maintenance chemotherapy, radiotherapy, or immunotherapy, and rehabilitation were considered always planned. Other scheduled or staged procedures were classified as planned only when a code for a potentially planned procedure was present, and the principal diagnosis was neither acute nor a complication of care; admissions for acute illness or treatment-related complications were classified as unplanned, even if anticancer therapy or a procedure was delivered during that admission. This approach was selected because ELECTIVE status alone may incompletely capture planned care, whereas the CMS algorithm provides a standardized method to improve the specificity of outcome classification and reduce potential misclassification in administrative data. The underlying CMS algorithm was developed and chart-validated in a general hospital population, and comparable claim-based classification principles are used in a cancer-specific unplanned-readmission measure, although direct validation in endometrial cancer is unavailable [14]. Within this framework, admissions primarily for scheduled chemotherapy or radiotherapy and qualifying non-acute procedures were excluded, whereas acute non-elective returns for treatment toxicity or other complications were retained as unplanned events. The remaining admissions were classified as unplanned readmissions.

#### 2.3.3. Candidate Predictors

Candidate variables were prespecified based on availability in the NRD and interpretability for nursing, including demographics and socioeconomic factors (age, primary payer PAY1, patient ZIP-code income quartile ZIPINC_QRTL), route of admission and presentation characteristics (whether via the emergency department HCUP_ED, whether weekend admission AWEEKEND, admission type ATYPE [Elective/Non-elective]), inpatient course (LOS, presence of a major operating room procedure PCLASS_ORPROC), discharge disposition DISPUNIFORM, and hospital-level characteristics (hospital region, teaching status, bed size, and ownership). In addition, the Elixhauser comorbidities (Refined, ICD-10-CM) and the CCSR tool were used to standardize the aggregation of comorbidities and diagnosis/procedure categories. The names, values, and sources of all variables are presented in Supplementary Table S2.

#### 2.3.4. Quantification of Comorbidities and Disease Severity

The Elixhauser Comorbidity Software v2025.1 Refined for ICD-10-CM was used to generate 38 binary comorbidity indicators and, according to the user guide, to calculate the 30-day readmission risk index and the in-hospital mortality risk index to reflect disease burden and baseline risk; diagnosis/procedure CCSR was used to achieve clinical category aggregation. To ensure consistency across 2016–2022, a single version of the mappings was applied to all years to batch-generate derived variables.

#### 2.3.5. Discharge Outcome and Start of Follow-Up

Alive discharge was identified using DISPUNIFORM and DIED; the start of follow-up was the day of discharge, and the endpoint was the first unplanned readmission or day 30 after discharge, whichever came first.

### 2.4. Data Processing and Quality Control

For each year, the core files, revisit files, and derived files were loaded and checked separately for consistency, and coding harmonization across years and variable name unification were performed. The specified versions of the PR CCSR and Elixhauser mappings were applied uniformly to diagnosis/procedure codes to ensure consistency across years. For time information, NRD_DaysToEvent and NRD_VisitLink were used together to compute the interval between hospitalizations, and same-day/next-day acute transfer records were merged according to NRD methods to avoid misclassifying readmissions. Missing data were handled according to a prespecified process: if the missing proportion of a given predictor was ≤5%, complete-case analysis was used; if >5% and ≤20%, multiple imputation with m = 10 was used (predictive mean matching for continuous variables and multinomial logistic regression for categorical variables), and estimates were combined according to Rubin’s rules; variables with >20% missingness were not included in model development. This threshold-based strategy was selected to balance methodological simplicity for variables with minimal missingness of and reduction in potential information loss for variables with moderate missingness. The predictor matrix included all prespecified discharge-available candidate predictors and the 30-day unplanned readmission outcome. In the present dataset, only ZIPINC_QRTL exceeded the 5% imputation threshold (8.01% missingness); therefore, its missing values were imputed using multinomial logistic regression with 10 iterations per imputed dataset. Convergence was assessed by visual inspection of trace plots. Thus, the difference in missing-data handling reflected the prespecified threshold rule rather than inconsistent processing, with all remaining predictors analyzed using complete-case methods because their missingness was minimal. All continuous variables were tested for linearity and, when necessary, modeled with restricted cubic splines.

### 2.5. Statistical Analysis

Data processing and analysis used R 4.3.2. Complex survey design used the survey package; regression modeling and splines used rms; penalized regression used glmnet; ROC curve calculations used pROC; decision curve analysis used rmda; and multiple imputation used mice. Using DISCWT together with NRD_STRATUM and HOSP_NRD, we constructed a complex survey-weighted design object and reported national-level characteristics of index hospitalizations and readmission incidence, along with their 95% confidence intervals. Group differences were evaluated using standardized mean differences rather than *p*-values to avoid overestimating statistical significance in this large cohort. Logistic regression was used to develop the model, with clinically and nursing-relevant variables prespecified for inclusion. To balance generalizability and interpretability, a two-step strategy was used: first, restricted cubic splines were applied to model the nonlinear effects of age and length of stay, using four-knot splines (age: 45.83, 56.94, 67.18, 79.42 years; LOS: 1.00, 1.96, 3.14, 6.82 days). Knot locations were prespecified according to the empirical quantiles of the development set and were treated as modeling parameters rather than clinical cut points. Second, without compromising the interpretability of nursing-relevant variables, LASSO regression was used for penalized variable selection to reduce redundancy and improve model parsimony. For the penalized step, all prespecified discharge-available candidate predictors were entered into a glmnet model matrix in the development set; age and LOS were entered as spline-expanded terms. The tuning parameter lambda was selected by 10-fold cross-validation using the minimum mean cross-validated binomial deviance (lamb-da.min rather than lambda.1se). Penalization was applied to individual model-matrix columns, including separate dummy-variable and spline-basis terms; grouped LASSO was not used. A prespecified hierarchical retention rule was applied: if any component term of a categorical or spline-modeled predictor had a non-zero coefficient at lambda.min, the parent predictor was retained, and all of its component terms were included in the subsequent survey-weighted multivariable logistic regression. The resulting parent predictors were retained based on non-zero coefficients at the selected lambda, and Wald *p*-values from the refitted model were not used for further variable deletion. Predictors with non-zero coefficients at the selected lambda were subsequently refitted in the survey-weighted multivariable logistic regression. All clinically and nursing-relevant candidate variables were prespecified before penalization; no separate pre-LASSO screening was performed, and no predictor whose component terms were all zero was re-entered after penalization. Multiple imputation was completed before penalized selection; because only ZIPINC_QRTL was imputed, the candidate predictor set itself did not vary across the imputed datasets. Penalized screening was run separately within each imputed dataset, and the retained parent-predictor set was identical across the 10 datasets; this common retained set, including all associated dummy-variable and spline-basis terms, was then refitted in the survey-weighted multivariable logistic regression, with coefficients combined according to Rubin’s rules. The glmnet step was used as an unweighted redundancy-reduction screen, after which the retained terms were refitted and evaluated within the complex survey-weighted design. Accordingly, AUC, Brier score, calibration intercept, calibration slope, and calibration curves were all derived from the survey-weighted models. All models were fitted in the development set (2016–2019). Within the development set, bootstrap (1000 times) was used to evaluate and correct optimism, and discrimination (AUC), Brier score, calibration intercept and slope, and quantile calibration curves were reported. In each bootstrap resample, the entire model-building pipeline, including model-matrix construction, 10-fold cross-validation for lambda.min, term-level LASSO screening, application of the hierarchical retention rule, and survey-weighted refitting were repeated, and optimism was estimated as the difference between bootstrap and test performance, and then subtracted from the apparent performance. Bootstrap resampling was performed at the hospital-cluster level, with HOSP_NRD clusters resampled with replacement within NRD_STRATUM; all discharges from sampled clusters were aggregated, and the survey design object was then rebuilt using DISCWT, NRD_STRATUM, and HOSP_NRD before performance evaluation so that validation preserved the NRD-stratified hospital-cluster structure. To evaluate clinical utility, prespecified risk thresholds of 5%, 10%, and 15% were used to calculate net benefit on decision curves. The 5% threshold approximated the cohort-level readmission incidence, whereas 10% and 15% represented progressively higher-risk levels at which increasingly intensive transitional-care interventions might be considered; consistent with decision-curve methodology, these thresholds were illustrative rather than universal clinical cutoffs [15].

Model stability was examined in the temporal validation cohorts: ① 2020–2021 as the pandemic-period validation; ② 2022 as the post-pandemic validation. In each validation cohort, AUC, Brier score, and calibration metrics were reported, and calibration curves were plotted. If systematic calibration drift occurred, recalibration with intercept-and-slope updating was performed, and the metrics before and after recalibration were presented side by side. Recalibration was implemented as logit(P) = α + β × LP_original, where α and β were re-estimated within each validation cohort. Because annual samples are mutually independent and cannot be tracked across years, extrapolation testing by temporal slicing was appropriate. Three preregistered sensitivity analyses were designed: ① an alternative “unplanned” definition (excluding elective admissions solely based on ELECTIVE = 0, without using the CMS algorithm); ② using 7-day readmission as the endpoint to test the predictability of early readmission; and ③ a broad definition of staging surgery (requiring only hysterectomy, without mandating lymph node procedures) to assess the impact of the definition of staging components on the model. Subgroup analyses reported model performance stratified by age (<65/≥65 years), insurance type, and surgical approach (abdominal/laparoscopic).

## 3. Results

### 3.1. Cohort Construction and Baseline Characteristics

**Figure 1:**
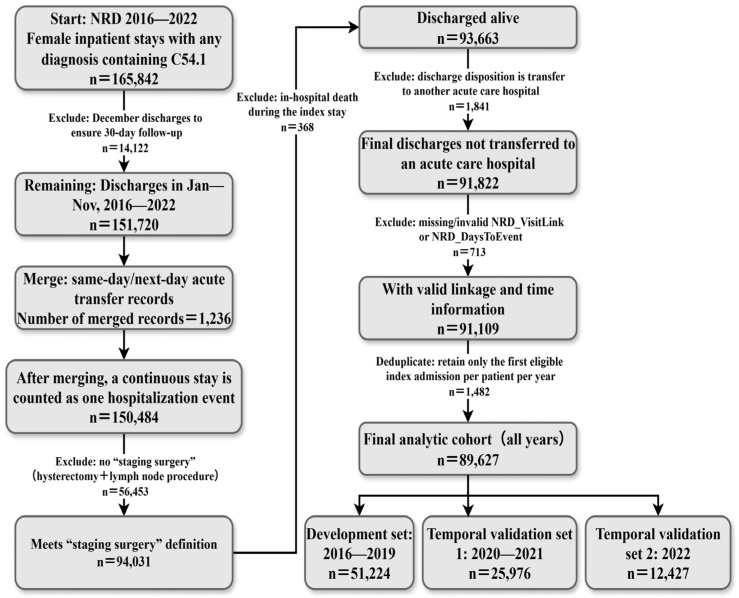
Cohort construction flowchart.

A total of 165,842 hospitalizations with suspected staging surgery for endometrial cancer in the NRD from 2016 to 2022 were included. After sequentially excluding December discharges, cases without staging surgery, in-hospital deaths, acute transfers, and records missing key linkage or time information, 89,627 analyzable index hospitalizations remained, of which 51,224 were assigned to the development set, 25,976 to temporal validation cohort 1 (2020–2021), and 12,427 to temporal validation cohort 2 (2022) (Figure 1). The corresponding unweighted observed numbers of 30-day unplanned readmission events were 2561 in the development set, 1374 in temporal validation cohort 1, and 613 in temporal validation cohort 2. After ZIPINC_QRTL imputation, each of the 10 imputed datasets initially contained 89,627 observations. Complete-case restriction for the remaining non-imputed predictors resulted in the same model-analysis sample of 85,013 observations with 4312 events in each imputed dataset, comprising 48,663 observations with 2441 events in the development set, 24,578 with 1293 events in temporal validation cohort 1, and 11,772 with 578 events in temporal validation cohort 2.

The three populations had similar baseline characteristics overall. Demographics, admission and presentation characteristics, discharge disposition (DISPUNIFORM), hospital-level characteristics, and comorbidity burden, represented by Elixhauser indices, showed good weighted baseline balance between the development and validation cohorts (all |SMD| < 0.10) (Table 1).

The overall missingness of candidate predictors was low (most items <1%), except for the patient ZIP code income quartile (ZIPINC_QRTL), which had an 8.01% missing rate. After multiple imputation with m = 10 for ZIPINC_QRTL, this variable yielded usable values for all 89,627 cases in the analytic cohort, with an FMI of 0.08 and a relative efficiency (RE) of 99.21% (Table 2).

### 3.2. Incidence and Temporal Trends of 30-Day Unplanned Readmission

**Figure 2:**
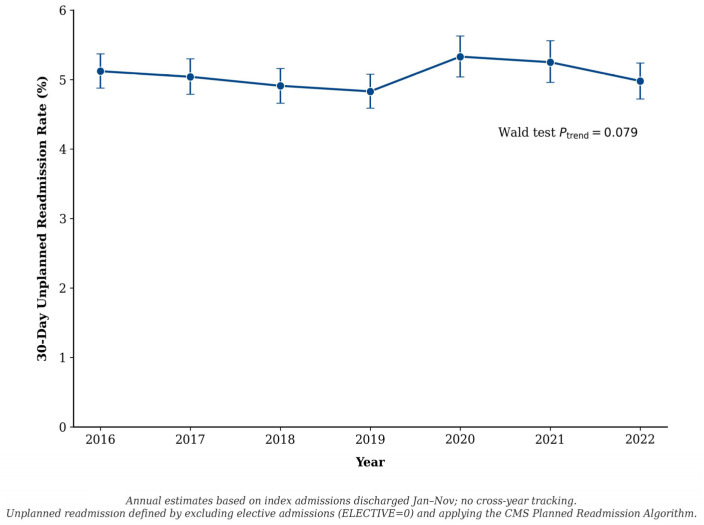
Annual Trends in Weighted 30-Day Unplanned Readmission Rates. Annual weighted 30-day unplanned readmission rates after endometrial cancer staging surgery, 2016–2022. Points represent annual complex survey-weighted estimates based on index hospitalizations discharged from January to November; error bars indicate 95% confidence intervals. No significant linear trend was observed across years (survey-weighted logistic regression with year entered as a continuous variable, *p* = 0.079).

Using complex survey-weighted logistic regression with year as a continuous variable to test linear trends, the 30-day unplanned readmission rate among patients undergoing staging surgery for endometrial cancer during 2016–2022 did not show a statistically significant linear trend (*p* = 0.079), suggesting that the overall readmission rate remained relatively stable during the study period (Figure 2).

### 3.3. Model Development and Internal Validation

In the development set, after 1000 bootstrap optimism corrections, the model showed moderate discrimination (AUC was 0.719) and good calibration (Brier 0.045, calibration intercept close to 0 [0.014], calibration slope close to 1 [0.955]), indicating good internal stability and a low risk of overfitting (Table 3).

**Figure 3:**
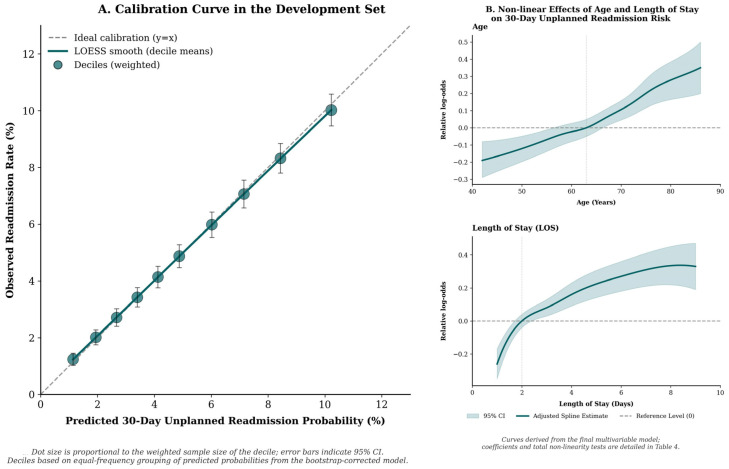
Model calibration and nonlinear associations in the development cohort. (**A**). Calibration curve of the final prediction model in the development cohort. The *x*-axis represents the predicted probability of 30-day unplanned readmission, and the *y*-axis represents the observed probability. The diagonal line indicates perfect calibration, and the calibration curve reflects the agreement between predicted and observed risks. (**B**). Restricted cubic spline analyses showing the nonlinear associations of age and length of stay (LOS) with the risk of 30-day unplanned readmission. The solid curves represent the estimated odds ratios across the observed ranges of age and LOS, and the shaded areas indicate the corresponding 95% confidence intervals. Restricted cubic splines were used to capture potential nonlinear relationships between predictors and readmission risk without assuming a constant linear effect.

Further decile-calibration analysis showed that predicted probabilities across risk deciles were generally consistent with observed 30-day unplanned readmission rates, and the overall calibration curve was smooth and close to the ideal diagonal, indicating good internal calibration (Figure 3A). The weighted multivariable logistic regression showed that, after controlling for other covariates, compared with discharge to home/self-care, discharge to home health or institutional care was associated with significantly increased odds of readmission (OR = 1.34–2.12). Non-elective admission (ATYPE, OR = 1.31), presentation via the emergency department (HCUP_ED, OR = 1.22), and a higher Elixhauser readmission risk index (per 1-point increase OR = 1.07) were all independently associated with readmission (all *p* < 0.05). Age and length of stay (LOS) showed significant nonlinear associations with 30-day unplanned readmission risk (both *p*_nonlin < 0.05) (Table 4). The complete model specification, including regression coefficients, restricted cubic spline parameters, and instructions for calculating individual risk estimates, is provided in Supplementary Table S3.

Specifically, increasing age was associated with a gradual increase in relative log-odds of readmission, with the median LOS (2 days) as the reference, risk was lower at 1 day, increased gradually after ≥3 days, and then plateaued (Figure 3B).

### 3.4. Temporal Validation and Model Recalibration

In the temporal validation cohorts within the NRD for 2020–2021 and 2022, the model AUCs were 0.708 and 0.713, and the Brier scores were 0.043–0.046, indicating overall stable discrimination (Table 5). After recalibration, the model showed improved calibration performance, with calibration intercepts and slopes moving closer to the ideal values. However, the AUC remained unchanged because recalibration adjusts the absolute risk estimates without affecting the relative ranking of predicted risks among individuals. Thus, discrimination was preserved while agreement between predicted and observed readmission risks improved (Table 5).

**Figure 4:**
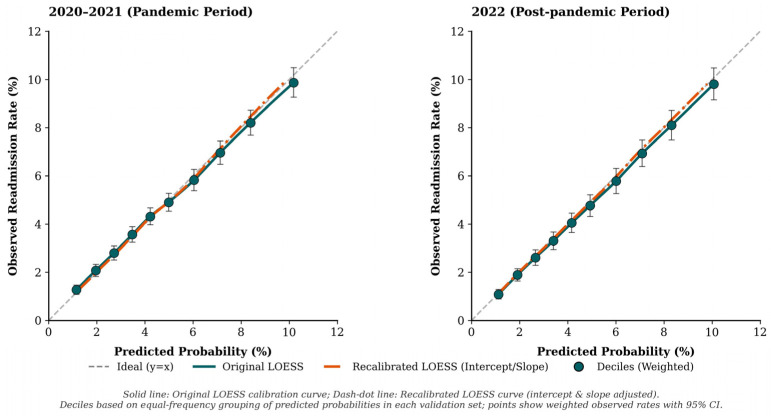
Temporal validation calibration curves after recalibration. Calibration curves of the original and recalibrated prediction models in the 2020–2021 (pandemic period) and 2022 (post-pandemic period) temporal validation cohorts. Recalibration was performed using logistic recalibration, updating the calibration intercept and slope. The *x*-axis represents the predicted probability of 30-day unplanned readmission, and the *y*-axis represents the observed readmission rate. The 45-degree diagonal line indicates ideal calibration. After recalibration, the curves demonstrated improved agreement between predicted and observed readmission risks, with calibration performance approaching the ideal condition of an intercept of 0 and a slope of 1.

The original model in 2020–2021 showed slight underestimation in low-risk deciles and slight overestimation in high-risk deciles, whereas in 2022 it showed overall slight overestimation. After recalibration, adjusting only the intercept and slope, the calibration curves for both periods were clearly closer to the ideal line (intercept ≈ 0, slope ≈ 1), with markedly improved calibration (Figure 4).

### 3.5. Clinical Utility, Subgroup, and Sensitivity Analyses

**Figure 5:**
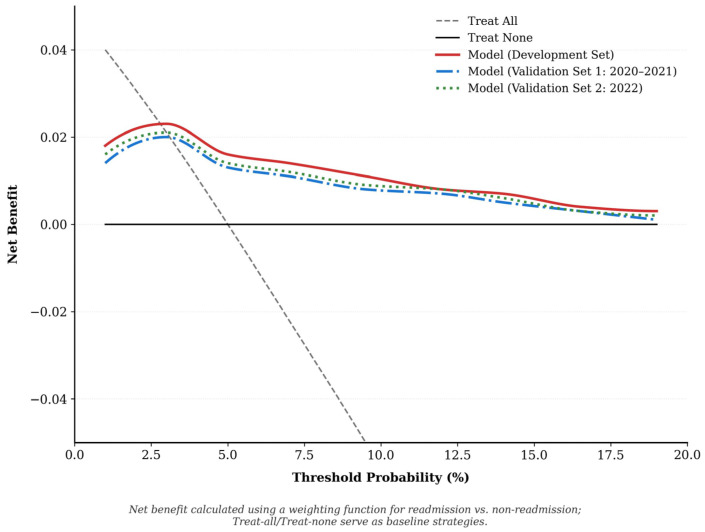
Decision Curve Analysis. Decision curve analysis was performed in the development set, Validation set 1 (2020–2021), and Validation set 2 (2022) to evaluate the net clinical benefit of the prediction model. Across the 0–20% threshold probability range, the model demonstrated positive net benefit at the prespecified thresholds of 5%, 10%, and 15% in all three datasets, indicating potential clinical usefulness across these decision thresholds. The *x*-axis represents the threshold probability of 30-day unplanned readmission, and the *y*-axis represents the net benefit. The “None” and “All” lines represent the strategies of assuming no patients or all patients would experience the outcome, respectively.

Within the 0–20% threshold range, the model showed positive net benefit at the prespecified thresholds of 5%, 10%, and 15% in the development set and both temporal validation cohorts, with consistent results across the three datasets, supporting potential clinical utility across these illustrative risk thresholds (Figure 5). Threshold-specific sensitivity, specificity, PPV, NPV, the number of patients classified as high risk per 100 discharges, and net benefit estimates for the model, treat-all, and treat-none strategies are presented in Supplementary Table S4.

In prespecified complex survey-weighted subgroup analyses, model performance was generally consistent across age strata, primary payer, and surgical approach: AUC 0.699–0.721, Brier score 0.042–0.047, and calibration slope close to 1 (approximately 0.943–0.987), indicating good stability of discrimination and calibration across different populations (Table 6). In sensitivity analyses in the development set, when defining unplanned readmission using only ELECTIVE = 0 (S1), using 7-day unplanned readmission as the endpoint (S2), or applying a broad definition of staging surgery (requiring only hysterectomy) (S3), the model AUC remained 0.712–0.729, Brier score 0.024–0.046, and calibration slope close to 1 (0.947–0.968), with small differences from the main analysis, indicating overall stable discrimination and calibration performance (Table 7).

## 4. Discussion

During the study period, the 30-day unplanned readmission rate after staging surgery for endometrial cancer did not show a significant linear change, suggesting that this outcome is driven more by relatively constant patient frailty, perioperative complication risk, and the quality of post-discharge transitional care management, rather than short-term structural fluctuations at the annual level [16]. National weighted estimates indicate that this burden is widespread across regions and hospital types, making readmission a stable target for nursing quality improvement and a potential focus for standardized risk assessment and stratified follow-up. This relatively stable baseline risk may inform planning of follow-up frequency and post-discharge services [17,18]. The development set and the two temporal validation cohorts were well comparable in demographics, admission and presentation characteristics, discharge disposition, hospital-level characteristics, and comorbidity burden, making temporal validation closer to the scenario of extrapolation across years in clinical practice and reducing the likelihood that changes in case mix would confound subsequent performance differences. Missingness in key predictors was generally limited, with missingness in socioeconomic indicators concentrated in the income quartile, and information loss after imputation was small, supporting the use of the socioeconomic dimension as a contextual covariate for discharge-time risk stratification without materially reducing estimation stability. Prior administrative database studies have reported similar postoperative readmission levels in gynecologic oncology and have indicated that fluctuations are mainly related to perioperative pathways and discharge resource allocation [19,20]. Prediction modeling methodology also emphasizes that temporal validation within the NRD should be based on a stable case mix and transparent handling of missing data; these features are prerequisites for interpreting temporal model stability. Weighted multivariable analysis indicates that discharge disposition, admission acuity, and comorbidity burden constitute the main risk axes for 30-day unplanned readmission after surgery, and the effects of age and length of stay (LOS) on risk are nonlinear. Discharge to home health or institutional care may indicate insufficient functional reserve, increased care needs, and limited social support; the longer chain of care handoffs makes medication reconciliation, symptom monitoring, and follow-up coordination more prone to problems [21]. Therefore, such patients require nursing-led discharge-readiness assessment, standardized health education, symptom-scale monitoring, early follow-up, and cross-institutional information handoff to reduce the risk of avoidable readmissions [22]. Presentation via the emergency department and non-elective admission reflect more pronounced perioperative physiologic instability or concomitant acute problems. There is a higher risk of readmission in the short term after discharge due to infection, bleeding, thrombosis, urinary complications, or poor pain control, and the window for nursing intervention should be moved earlier to inpatient outcome management and proactive contact within the first week after discharge [23,24]. The nonlinear relationships of age and LOS suggest that risk does not increase uniformly with these variables; the surge at older ages is more consistent with threshold effects of frailty and multimorbidity, and prolonged LOS within a certain range is more likely to reflect delayed recovery or complication burden, which should trigger higher-intensity transitional care pathways [25]. Prior studies on perioperative quality and transitional care have generally regarded discharge disposition, acute admission characteristics, and comorbidities as stable predictors of readmission and have emphasized that stratified follow-up and nursing handoffs for high-risk populations can reduce readmissions [26]. The risk profile in this study is consistent with that evidence and, together with the nonlinear information, further supports use of the model to inform risk-stratified nurse-led transitional care rather than uniform interventions.

After bootstrap optimism correction, the model showed stable discrimination and near-ideal calibration in the development set, suggesting that the predictors can effectively rank readmission risk at the individual level and that overfitting is controllable. The discrimination achieved by the present model (AUC 0.708–0.719) should be interpreted as moderate rather than high. However, this performance is comparable to that reported by many readmission prediction models developed using administrative or routinely collected healthcare data, with AUC values typically ranging from 0.65 to 0.75. Because the present model was designed as a discharge-time administrative-data tool using variables available across healthcare settings, rather than detailed tumor-, laboratory-, or procedure-specific variables, a moderate level of discrimination represents a reasonable balance between predictive performance, interpretability, and practical applicability. Although more complex machine learning approaches, such as Random Forest, Gradient Boosting, XGBoost, or Elastic Net, may provide potential improvements in predictive performance, the present study prioritized model interpretability, transparent risk estimation, and practical implementation. Accordingly, the decision not to conduct head-to-head comparisons with alternative machine learning prediction algorithms was a deliberate methodological choice aligned with the study objective. A logistic regression-based framework allows for straightforward calculation of individual risk probabilities, easier recalibration across different healthcare settings, and clearer interpretation of predictor contributions. These characteristics are particularly relevant for a discharge-time nursing risk stratification tool, where transparency and usability are essential for integration into routine transitional care workflows. Future studies should compare this interpretable approach with alternative machine learning algorithms to determine whether increased model complexity provides meaningful improvements in discrimination, calibration, and clinical utility. When extrapolating the same model by year to the pandemic-period and post-pandemic populations, discrimination remained consistent, indicating that the risk-ranking relationships were transportable across periods. The study period overlapped with the COVID-19 pandemic, during which substantial changes occurred in healthcare utilization, hospital admission practices, outpatient access, and post-discharge care pathways. These changes may have influenced short-term readmission patterns by altering patients’ healthcare-seeking behaviors, thresholds for emergency evaluation, availability of follow-up services, and transitional care processes. Although we did not directly incorporate COVID-19 infection status or pandemic-related healthcare policy changes into the model, the consistent discrimination and calibration performance observed across the 2020, 2021, and 2022 temporal validation cohorts suggest that the identified risk factors remained clinically relevant despite these system-level variations. Future studies incorporating pandemic-specific factors and external validation across diverse healthcare systems are warranted to further evaluate the model’s adaptability. The discrimination observed in the present study (AUC 0.708–0.719) should be interpreted as moderate and is broadly consistent with previously published oncology readmission prediction models. For example, the Cancer READMIT score, developed for patients with solid tumor malignancies, and other gynecologic oncology readmission prediction tools have also demonstrated moderate discrimination rather than excellent performance [9,10]. This reflects the multifactorial nature of postoperative readmissions and the inherent difficulty of predicting these events using routinely available healthcare data. Although direct comparison is limited by differences in patient populations, surgical settings, predictor availability, and outcome definitions, these findings indicate that moderate discrimination may represent a realistic performance level for clinically applicable readmission prediction models. Predicted and observed values across risk deciles were generally concordant. After recalibration, the curves were closer to the ideal line, indicating that the main differences stemmed from small shifts in baseline risk. Calibration could be restored by intercept-and-slope updating alone, implying low maintenance cost and allowing for rolling updates within quality improvement cycles without rebuilding the variable set [27]. This approach could generate risk tiers at discharge to inform differentiated follow-up and referral decisions. The prespecified thresholds of 5%, 10%, and 15% should be interpreted as illustrative decision thresholds for local transitional pathway development, rather than as fixed, universal clinical cutoffs. For example, patients with lower predicted risk may receive routine discharge education, whereas patients exceeding higher risk thresholds may be considered for earlier follow-up and intensified transitional care coordination, depending on local resources, workflow capacity, and clinical priorities. Decision curves consistently yielded positive net benefit within the prespecified threshold range, suggesting potential use of the model to identify patients for consideration of enhanced discharge readiness, medication reconciliation, symptom monitoring, and early follow-up, thereby shifting nursing resources from universal coverage to precise targeting. Although discrimination was moderate, decision curve analysis demonstrated consistent positive net benefit across clinically relevant threshold probabilities in both development and temporal validation cohorts. These findings suggest that the model may still provide meaningful clinical value by supporting the identification of patients who could benefit from enhanced discharge planning, early follow-up, medication reconciliation, and other nursing-led transitional care interventions. Therefore, the utility of the model should be evaluated not only by its AUC but also by its ability to facilitate more efficient allocation of transitional care resources. Metrics were consistent across different ages, primary payer categories, and surgical approaches, indicating robustness to heterogeneous populations [28]. Sensitivity analyses under alternative outcome definitions and cohort delineations maintained similar performance; shorter follow-up endpoints were more readily identified, suggesting that very early readmissions are more influenced by the inpatient course and discharge transitions and are better suited as priority targets for transitional care interventions [29,30]. Prior prediction modeling methodology emphasizes joint reporting of temporal validation, calibration, and clinical utility, and notes that administrative-data models often require recalibration to adapt to new periods; the present findings support temporal robustness within the NRD but do not establish transportability across healthcare systems. From an implementation perspective, this model could be integrated into the discharge planning workflow as an automated risk assessment tool within electronic health record systems or as a standalone clinical calculator. Nurses could use the predicted risk estimate to support individualized transitional care planning before discharge. Patients identified as higher risk may benefit from intensified interventions, including early post-discharge follow-up, medication reconciliation, reinforcement of discharge education, symptom surveillance, and coordination of outpatient care. However, the optimal thresholds for assigning different levels of follow-up intensity require prospective validation and should be adapted to local healthcare resources and nursing capacity. Therefore, the current model should be considered a discharge-time decision-support tool that may support nurse-led transitional care planning rather than a replacement for clinical judgment.

### Limitations

Several limitations should be acknowledged. First, the NRD is a hospital administrative database and lacks detailed oncologic and perioperative information, including FIGO staging, pathologic grading, histologic subtype, intraoperative findings, estimated blood loss, operative time, key laboratory indicators, imaging information, and postoperative complication severity. It also does not include nursing process variables such as quality of discharge education, nursing staffing, and follow-up adherence. Therefore, the model mainly relies on proxy indicators available in the database, including comorbidities, presentation characteristics, inpatient course, and discharge disposition, which may limit its ability to identify specific types of complications or delineate causal pathways involving modifiable factors. Furthermore, although the Elixhauser Readmission Risk Index was included as a standardized measure of comorbidity burden, the present model was not designed as a nursing-only prediction model. Therefore, the incremental predictive value of a model that excludes the Elixhauser index and relies exclusively on nursing-specific variables was not evaluated separately. Future studies should investigate whether a nursing-focused model can provide comparable predictive performance while improving clinical interpretability.

Second, although missingness among candidate predictors was generally low, residual bias due to missing data cannot be entirely ruled out because the underlying mechanism of missingness cannot be fully verified in administrative databases. Complete-case analysis for variables with minimal missingness may theoretically introduce bias if missingness is informative; however, the proportion of missing observations for these variables was very small. The only predictor with moderate missingness was ZIPINC_QRTL (8.01%), which was addressed using multiple imputation, and the high relative efficiency suggests that imputation uncertainty was limited.

Third, NRD supports patient linkage only within the same calendar year and cannot track patients across different years; therefore, cross-state readmissions may be missed. Although this study reduced the risk of incomplete follow-up by limiting discharges to January–November, the true readmission burden may still be underestimated. The definition of unplanned readmission relied on the NRD-derived ELECTIVE variable together with the CMS Planned Readmission Algorithm, a widely used approach in administrative database research. Nevertheless, residual outcome misclassification remains possible because some planned admissions may be classified as unplanned, and some true unplanned readmissions may be excluded. The exact magnitude of this misclassification cannot be directly quantified because no gold-standard classification of unplanned readmission is available within the NRD. Because both the primary definition and the ELECTIVE = 0 sensitivity definition rely on administrative coding, their classification errors may be correlated, and the direction and magnitude of any resulting bias cannot be determined. Accordingly, the similar discrimination and calibration observed in the sensitivity analysis indicate robustness to an alternative operational definition, but do not validate definition or demonstrate that outcome misclassification was limited.

Fourth, staging surgery and surgical approach were obtained by mapping ICD-10-PCS codes and classification tools; therefore, coding differences and variations in hospital documentation practices may introduce misclassification. In addition, although temporal validation within the NRD demonstrated stable model performance across different time periods, an independent external validation cohort from other institutions or geographic regions was not available in the present study. Therefore, the generalizability and transportability of the model require further evaluation in diverse healthcare settings. Because the model was developed using data from the U.S. healthcare system, differences in healthcare organization, payment structures, discharge-planning practices, nursing resources, and post-discharge follow-up systems may affect its performance when applied to other countries and low-resource settings. Therefore, the model should not be directly implemented in external populations without prior external validation, local recalibration, and, if necessary, model updating. In resource-limited settings, where detailed clinical information may be less readily available, the model may provide a framework for risk stratification using routinely collected administrative variables; however, its predictive performance and clinical utility require confirmation in the local healthcare context.

Finally, although the present model demonstrated stable discrimination and calibration, the absence of detailed clinical variables may limit further improvement in predictive performance. Incorporating tumor characteristics, surgical details, laboratory findings, and perioperative information from linked electronic health records or clinical registries may enhance prediction and provide greater insight into disease-specific mechanisms underlying readmission. Future studies should evaluate a two-tier framework comprising a discharge-based administrative model for real-time nursing decision support and an enhanced clinical model integrating granular clinical information to determine the incremental value of additional predictors.

## 5. Conclusions

During the study period, 30-day unplanned readmission rates remained stable, suggesting that this outcome may be influenced by relatively persistent patient-level vulnerability and transitional care-related factors. The discharge-time administrative data-based risk prediction model developed using the NRD demonstrated moderate but stable discrimination and good calibration in the development and temporal validation cohorts, with minor temporal drift corrected through intercept-and-slope updating. The model identified patients at higher risk of readmission based on discharge-available characteristics, including discharge disposition, emergency or non-elective admission, and comorbidity burden. Decision curve analyses suggested potential clinical utility across prespecified risk thresholds, and subgroup and sensitivity analyses supported model robustness. This model represents a candidate discharge-time decision-support tool that may support nurse-led transitional care planning; however, further local recalibration and prospective validation are required before implementation in diverse clinical settings.

## Figures and Tables

**Table 1:** Baseline characteristics of index hospitalizations (weighted).

Variable	Development Set (Weighted) n = 51,224	Validation Cohort 1: 2020–2021 (Weighted) n = 25,976	Validation Cohort 2: 2022 (Weighted) n = 12,427	SMD (Development vs. Validation 1)	SMD (Development vs. Validation 2)
Demographics					
Age (years)	62.38 ± 10.94	63.12 ± 11.08	62.07 ± 10.81	−0.067	0.029
Female (%)	100	100	100	NA	NA
Socioeconomic and payer					
PAY1: Medicare (%)	56.83	58.21	56.11	−0.028	0.015
PAY1: Medicaid (%)	8.74	9.13	8.59	−0.014	0.005
PAY1: Private including HMO (%)	28.92	27.14	29.47	0.04	−0.012
PAY1: Self-pay/No charge (%)	2.61	2.47	2.68	0.009	−0.004
PAY1: Other (%)	2.9	3.05	3.15	−0.009	−0.015
ZIPINC_QRTL: Q1 (%)	20.63	21.02	20.12	−0.01	0.013
ZIPINC_QRTL: Q2 (%)	24.87	25.21	24.66	−0.008	0.005
ZIPINC_QRTL: Q3 (%)	26.91	26.39	27.08	0.012	−0.004
ZIPINC_QRTL: Q4 (%)	27.59	27.38	28.14	0.005	−0.012
Admission and presentation characteristics					
HCUP_ED (%)	14.37	15.28	13.92	−0.026	0.013
AWEEKEND (%)	4.31	4.76	4.19	−0.022	0.006
ATYPE: Elective (%)	84.72	82.94	85.51	0.048	−0.022
ATYPE: Non-elective (%)	15.28	17.06	14.49	−0.048	0.022
Inpatient course					
Length of stay LOS (days)	2.64 ± 1.92	2.51 ± 1.88	2.47 ± 1.81	0.068	0.091
Major operating room procedure PCLASS_ORPROC (%)	98.36	98.11	98.49	0.019	−0.01
Discharge disposition DISPUNIFORM					
Home/self-care (%)	81.93	82.61	83.24	−0.018	−0.035
Home health (%)	12.47	11.88	11.56	0.018	0.028
Short-term hospital (%)	0.46	0.53	0.42	−0.01	0.006
Skilled nursing/intermediate care facility (%)	4.71	4.61	4.36	0.005	0.017
Against medical advice (%)	0.43	0.37	0.42	0.01	0.002
Hospital-level					
Region: Northeast (%)	19.37	19.92	19.64	−0.014	−0.007
Region: Midwest (%)	24.51	24.23	24.19	0.007	0.007
Region: South (%)	36.98	36.41	36.27	0.012	0.015
Region: West (%)	19.14	19.44	19.9	−0.008	−0.019
Teaching: Teaching (%)	63.28	65.44	64.12	−0.045	−0.017
Teaching: Non-teaching (%)	36.72	34.56	35.88	0.045	0.017
Bed size: Small (%)	10.84	11.23	10.61	−0.012	0.007
Bed size: Medium (%)	22.97	22.14	21.84	0.02	0.027
Bed size: Large (%)	66.19	66.63	67.55	−0.009	−0.029
Ownership: Government nonfederal (%)	6.91	7.14	6.78	−0.009	0.005
Ownership: Private nonprofit (%)	78.27	77.63	78.41	0.015	−0.003
Ownership: Private investor-owned (%)	14.82	15.23	14.81	−0.011	<0.001
Comorbidity burden (Elixhauser indices)					
Elixhauser readmission risk index	4.83 ± 3.17	4.97 ± 3.24	4.72 ± 3.05	−0.044	0.035
Elixhauser in-hospital mortality risk index	3.11 ± 2.26	3.23 ± 2.31	3.07 ± 2.18	−0.053	0.018
30-day unplanned readmission, unweighted n (%)	2561 (4.97)	1374 (5.29)	613 (4.98)	—	—

Note: Cohort sample sizes and event counts are unweighted observed counts, whereas means, standard deviations, percentages, and SMDs are survey-weighted. SMD is the weighted standardized mean difference (|SMD| ≥ 0.10 considered a substantive difference). For continuous variables, SMD uses the mean difference divided by the pooled SD; for binary or categorical variables, SMD is standardized by the pooled variance in the difference in proportions. Elective indicates NRD-derived ELECTIVE = 1; Non-elective indicates ELECTIVE = 0. Elixhauser indices are generated using the ICD-10-CM Refined tools; higher values indicate higher risk.

**Table 2:** Variable missingness and missing-data-handling summary.

Variable	Missing n	Missing %	Imputation Method	Effective Sample Size After Imputation n_eff
Age	73	0.08	Complete-case	89,554
PAY1	641	0.72	Complete-case	88,986
ZIPINC_QRTL	7183	8.01	Multinomial logistic regression	89,627
HCUP_ED	214	0.24	Complete-case	89,413
AWEEKEND	91	0.1	Complete-case	89,536
ATYPE	329	0.37	Complete-case	89,298
LOS	118	0.13	Complete-case	89,509
PCLASS_ORPROC	647	0.72	Complete-case	88,980
DISPUNIFORM	504	0.56	Complete-case	89,123
Hospital region	77	0.09	Complete-case	89,550
Teaching status	202	0.23	Complete-case	89,425
Bed size	293	0.33	Complete-case	89,334
Ownership	431	0.48	Complete-case	89,196
Elixhauser readmission risk index	589	0.66	Complete-case	89,038
Elixhauser in-hospital mortality risk index	603	0.67	Complete-case	89,024

Note: Multiple imputation was performed using m = 10 imputations. Only ZIPINC_QRTL underwent multiple imputation because it was the only predictor with missingness >5% according to the prespecified rule. The FMI was 0.08 and the RE was 99.21%. RE was calculated as 1/(1 + FMI/m) × 100%. Variables with missingness ≤5% were handled using complete-case analysis; variables with 5–20% missingness underwent multiple imputation with m = 10; variables with >20% missingness were excluded from modeling. Each imputed dataset initially contained 89,627 observations; after restriction to complete cases for the non-imputed predictors, 85,013 observations with 4312 events were available for model analyses. The n_eff values are variable-specific available sample sizes and should not be interpreted as model-level complete-case counts.

**Table 3:** Internal validation performance in the development set.

Performance Metric	Apparent Estimate	Optimism	Optimism-Corrected Estimate (95% CI)
AUC	0.732	0.013	0.719 (0.709, 0.730)
Brier score	0.044	−0.001	0.045 (0.044, 0.046)
Calibration intercept	0.002	−0.012	0.014 (−0.008, 0.036)
Calibration slope	0.998	0.043	0.955 (0.936, 0.981)

Note: Performance metrics were derived from the survey-weighted development-set model and were optimism-corrected via bootstrap resampling, with reconstruction of the NRD survey design in each resample. The development-set sample size and event count were unweighted observed counts: n = 51,224, with 2561 30-day unplanned readmission events. The eligible development cohort included 51,224 observations with 2561 events, whereas the model-level complete-case analysis included 48,663 observations with 2441 events. All sample and event counts are unweighted observed counts.

**Table 4:** Effect sizes and tests for the final multivariable logistic regression model.

Variable and Reference Level	OR (95% CI)/Wald χ^2^ (*p*_nonlin)	*p*-Value
Age (restricted cubic splines)	χ^2^ = 28.435; *p*_nonlin = 0.012	0.001
Length of stay LOS (restricted cubic splines)	χ^2^ = 54.783; *p*_nonlin = 0.004	<0.001
Primary payer (reference = Private including HMO)		
Medicare	1.18 (1.08–1.29)	0.001
Medicaid	1.27 (1.12–1.44)	0.001
Self-pay/No charge	1.21 (1.02–1.43)	0.029
Other	1.09 (0.97–1.23)	0.143
ZIPINC_QRTL (reference = Q4 highest income)		
Q1	1.13 (1.04–1.23)	0.006
Q2	1.09 (1.01–1.18)	0.024
Q3	1.05 (0.98–1.13)	0.161
HCUP_ED (reference = no)	1.22 (1.12–1.34)	<0.001
AWEEKEND (reference = no)	1.06 (0.96–1.18)	0.241
Admission type ATYPE (reference = Elective)	1.31 (1.19–1.45)	<0.001
Major operating room procedure PCLASS_ORPROC (reference = 0)	0.98 (0.85–1.12)	0.734
Discharge disposition (reference = home/self-care)		
Home health	1.34 (1.23–1.46)	<0.001
Short-term hospital	1.76 (1.21–2.54)	0.003
Skilled nursing/intermediate care facility	2.12 (1.92–2.35)	<0.001
Against medical advice	1.61 (1.09–2.37)	0.017
Hospital region (reference = Northeast)		
Midwest	0.97 (0.90–1.05)	0.312
South	1.06 (1.01–1.13)	0.041
West	0.95 (0.88–1.03)	0.072
Teaching status (reference = Non-teaching)	1.05 (1.00–1.10)	0.048
Bed size (reference = Small)		
Medium	0.96 (0.86–1.07)	0.214
Large	1.03 (0.92–1.16)	0.575
Ownership (reference = Private nonprofit)		
Government nonfederal	1.08 (0.99–1.19)	0.089
Private investor-owned	1.04 (0.96–1.13)	0.251
Elixhauser readmission risk index (per 1-point)	1.07 (1.05–1.08)	<0.001
Elixhauser in-hospital mortality risk index (per 1-point)	1.05 (1.02–1.07)	0.002

Note: *p*_nonlin is the joint test for the nonlinear terms.

**Table 5:** Temporal validation performance.

Metric	2020–2021 Original Validation	2020–2021 After Recalibration	2022 Original Validation	2022 After Recalibration
AUC	0.708 (0.694–0.722)	0.708 (0.694–0.722)	0.713 (0.697–0.729)	0.713 (0.697–0.729)
Brier score	0.046 (0.045–0.047)	0.045 (0.044–0.046)	0.044 (0.043–0.045)	0.043 (0.042–0.044)
Calibration intercept	0.083 (0.048–0.117)	0.004 (−0.018–0.026)	−0.031 (−0.062–−0.001)	0.002 (−0.019–0.023)
Calibration slope	0.927 (0.883–0.971)	0.999 (0.957–1.044)	0.962 (0.918–1.008)	1.003 (0.961–1.049)

Note: Performance metrics were derived under the survey-weighted design. The eligible validation cohorts included 25,976 observations with 1374 events for 2020–2021 and 12,427 observations with 613 events for 2022; the corresponding model-level complete-case samples included 24,578 observations with 1293 events and 11,772 observations with 578 events, respectively. All sample and event counts are unweighted observed counts. Recalibration adjusted only the intercept and slope and did not change the model variable composition.

**Table 6:** Prespecified subgroup performance.

Subgroup	Unweighted Complete Cases, n	Unweighted Events, n	AUC (95% CI)	Brier (95% CI)	Calibration Slope (95% CI)
Age < 65 years	38,265	1508	0.707 (0.691–0.722)	0.043 (0.042–0.045)	0.962 (0.918–1.011)
Age ≥ 65 years	46,748	2804	0.716 (0.702–0.729)	0.046 (0.045–0.047)	0.973 (0.932–1.030)
PAY1 = Medicare	48,308	2717	0.714 (0.700–0.728)	0.046 (0.045–0.047)	0.961 (0.917–1.012)
PAY1 = Medicaid	7458	368	0.699 (0.677–0.721)	0.047 (0.046–0.049)	0.943 (0.885–1.012)
PAY1 = Private including HMO	24,417	931	0.718 (0.700–0.735)	0.043 (0.042–0.045)	0.972 (0.930–1.026)
PAY1 = Self-pay/No charge	2203	136	0.704 (0.668–0.740)	0.045 (0.043–0.048)	0.955 (0.889–1.039)
PAY1 = Other	2627	160	0.705 (0.671–0.739)	0.046 (0.044–0.049)	0.958 (0.893–1.041)
Surgical approach = abdominal	30,635	1889	0.721 (0.704–0.738)	0.047 (0.046–0.048)	0.987 (0.945–1.041)
Surgical approach = laparoscopic	54,378	2423	0.706 (0.692–0.720)	0.042 (0.041–0.043)	0.949 (0.906–1.001)

Note: Subgroup sample sizes and event counts are unweighted model-level complete-case counts; AUC, Brier score, and calibration slope were estimated under the survey-weighted design. Surgical approach was classified using the ICD-10-PCS mapping. Subgroup AUC/Brier/slope are reported only to assess robustness across populations. To avoid redundancy, the calibration intercept is not reported (baseline risk differences influence the intercept and can be misleading in subgroup comparisons).

**Table 7:** Sensitivity analysis results.

Scenario	AUC (95% CI)	Brier (95% CI)	Calibration Slope (95% CI)	ΔAUC, ΔBrier, ΔSlope
S1	0.712 (0.701–0.724)	0.046 (0.045–0.047)	0.947 (0.928–0.971)	−0.007, +0.001, −0.008
S2	0.729 (0.711–0.747)	0.024 (0.023–0.025)	0.968 (0.939–1.003)	+0.010, −0.021, +0.013
S3	0.714 (0.702–0.727)	0.046 (0.045–0.047)	0.948 (0.930–0.976)	−0.005, +0.001, −0.007

Note: S1 defines “unplanned” using only ELECTIVE = 0; S2 uses 7-day unplanned readmission as the endpoint; S3 applies a broad definition of staging surgery (requiring only hysterectomy). Δ was calculated relative to the optimism-corrected main analysis performance in the development set.

## Data Availability

The data presented in this study are openly available in Healthcare Cost and Utilization Project (HCUP) Nationwide Readmissions Database (NRD) (https://www.hcup-us.ahrq.gov/nrdoverview.jsp) (accessed on 15 January 2026).

## Supplementary Materials

The following supporting information can be downloaded at: https://www.mdpi.com/article/10.3390/healthcare14152249/s1, File S1: TRIPOD Checklist: Prediction Model Development and Validation; Table S1: ICD-10-PCS Codes Used to Identify Hysterectomy and Lymph Node Procedures; Table S2: Study Variables Derived from the Nationwide Readmissions Database (NRD); Table S3: Full specification of the final multivariable logistic regression model for 30-day unplanned readmission after endometrial cancer staging surgery; Table S4: Threshold-Specific Operating Characteristics, Net Benefit, and Illustrative Nursing Pathways of the Prediction Model.

## References

[1] Kalampokas E., Giannis G., Kalampokas T., Papathanasiou A.-A., Mitsopoulou D., Tsironi E., Triantafyllidou O., Gurumurthy M., Parkin D.E., Cairns M. (2022). Current Approaches to the Management of Patients with Endometrial Cancer. Cancers.

[2] Miguet C., Jauffret C., Zemmour C., Boher J.-M., Sabiani L., Houvenaeghel G., Blache G., Brun C., Lambaudie E. (2023). Enhanced Recovery after Surgery and Endometrial Cancers: Results from an Initial Experience Focused on Elderly Patients. Cancers.

[3] Sauro K.M., Smith C., Ibadin S., Thomas A., Ganshorn H., Bakunda L., Bajgain B., Bisch S.P., Nelson G. (2024). Enhanced Recovery After Surgery Guidelines and Hospital Length of Stay, Readmission, Complications, and Mortality: A Meta-Analysis of Randomized Clinical Trials. JAMA Netw. Open.

[4] Li R.D., Joung R.H.-S., Chung J.W., Holl J., Bilimoria K.Y., Merkow R.P. (2024). Divergent Trends in Postoperative Length of Stay and Postdischarge Complications over Time. Jt. Comm. J. Qual. Patient Saf..

[5] Riveros C., Ranganathan S., Shah Y.B., Huang E., Xu J., Geng M., Melchiode Z., Hu S., Miles B.J., Esnaola N. (2023). Postoperative Discharge Destination Impacts 30-Day Outcomes: A National Surgical Quality Improvement Program Multi-Specialty Surgical Cohort Analysis. J. Clin. Med..

[6] Sakashita C., Endo E., Ota E., Oku H. (2025). Effectiveness of nurse-led transitional care interventions for adult patients discharged from acute care hospitals: A systematic review and meta-analysis. BMC Nurs..

[7] Ram S., Gilboa I., Ariel G., Sabag D.N., Grisaru D., Michaan N., Laskov I., Lavie M. (2026). Risk factors for hospital readmission following staging surgery for endometrial cancer. Eur. J. Obstet. Gynecol. Reprod. Biol..

[8] Morell A., Samborski A., Williams D., Anderson E., Kittel J., Thevenet-Morrison K., Wilbur M. (2023). Calculating surgical readmission rates in gynecologic oncology: The impact of patient factors. Gynecol. Oncol..

[9] Manzano J.-G.M., Lin H., Zhao H., Halm J., Suarez-Almazor M.E. (2022). Derivation and Validation of the Cancer READMIT Score: A Readmission Risk Scoring System for Patients With Solid Tumor Malignancies. JCO Oncol. Pract..

[10] Lightfoot M.D.S., Felix A.S., Bishop E.E., Henderson A.P., Vetter M.H., Salani R., O’Mallley D.M., Bixel K.L., Cohn D.E., Fowler J.M. (2022). Who will be readmitted? Evaluation of the laparoscopic hysterectomy readmission score in a gynecologic oncology population undergoing robotic-assisted hysterectomy. Gynecol. Oncol..

[11] Tran P.T., Slayton W.B., Dalal M., Brown J. (2020). Incidence and Risk Factors for 30-Day Readmission after Inpatient Chemotherapy among Acute Lymphoblastic Leukemia Patients. Healthcare.

[12] Collins G.S., Reitsma J.B., Altman D.G., Moons K.G.M. (2015). Transparent reporting of a multivariable prediction model for individual prognosis or diagnosis (TRIPOD): The TRIPOD statement. BMJ (Clin. Res. Ed.).

[13] HCUP HCUP Nationwide Readmissions Database (NRD). Healthcare Cost and Utilization Project (HCUP). Agency for Healthcare Research and Quality. https://hcup-us.ahrq.gov/db/vars/key_nrd/nrdnote.jsp.

[14] Horwitz L.I., Grady J.N., Cohen D.B., Lin Z., Volpe M., Ngo C.K., Masica A.L., Long T., Wang J., Keenan M. (2015). Development and validation of an algorithm to identify planned readmissions from claims data. J. Hosp. Med..

[15] Vickers A.J., Elkin E.B. (2006). Decision curve analysis: A novel method for evaluating prediction models. Med. Decis. Mak. Int. J. Soc. Med. Decis. Mak..

[16] Melamed A., Katz Eriksen J.L., Hinchcliff E.M., Worley M.J.J., Berkowitz R.S., Horowitz N.S., Muto M.G., Urman R.D., Feltmate C.M. (2016). Same-Day Discharge After Laparoscopic Hysterectomy for Endometrial Cancer. Ann. Surg. Oncol..

[17] Saeed S., Patel R., Odeyemi R. (2022). Calibrating Readmission Risk Prediction Models for Determining Post-discharge Follow-up Timing. J. Community Hosp. Intern. Med. Perspect..

[18] Balasubramanian I., Andres E.B., Malhotra C. (2025). Outpatient Follow-Up and 30-Day Readmissions: A Systematic Review and Meta-Analysis. JAMA Netw. Open.

[19] Cappuccio S., Li Y., Song C., Liu E., Glaser G., Casarin J., Grassi T., Butler K., Magtibay P., Magrina J.F. (2021). The shift from inpatient to outpatient hysterectomy for endometrial cancer in the United States: Trends, enabling factors, cost, and safety. Int. J. Gynecol. Cancer Off. J. Int. Gynecol. Cancer Soc..

[20] Wield A.M., Cohen M.G., Toal C.T., Kulinsky M., Holder-Murray J.M., Esper S.A., Boisen M.M., Courtney-Brooks M.B., Taylor S.E. (2022). Same-day Hospital Discharge after Minimally Invasive Hysterectomy in a Gynecologic Oncology Practice: Feasibility, Safety, Predictors of Admission, and Adverse Outcomes. J. Minim. Invasive Gynecol..

[21] Ravn-Nielsen L.V., Bjørk E., Nielsen M., Galsgaard S., Pottegård A., Lundby C. (2024). Challenges related to transitioning from hospital to temporary care at a skilled nursing facility: A descriptive study. Eur. Geriatr. Med..

[22] Becker C., Zumbrunn S., Beck K., Vincent A., Loretz N., Müller J., Amacher S.A., Schaefert R., Hunziker S. (2021). Interventions to Improve Communication at Hospital Discharge and Rates of Readmission: A Systematic Review and Meta-analysis. JAMA Netw. Open.

[23] Kohut A., Earnhardt M.C., Cuccolo N.G., Kim C.-S., Song M., Girda E., De Meritens A.B., Stephenson R., Balica A., Leiser A. (2020). Evaluating unplanned readmission and prolonged length of stay following minimally invasive surgery for endometrial cancer. Gynecol. Oncol..

[24] Penn C.A., Morgan D.M., Rice L.W., Harris J.A., Rauh-Hain J.A., Uppal S. (2016). Timing of and Reasons for Unplanned 30-Day Readmission After Hysterectomy for Benign Disease. Obstet. Gynecol..

[25] Sia T.Y., Wen T., Cham S., Friedman A.M., Wright J.D. (2021). The effect of frailty on postoperative readmissions, morbidity, and mortality in endometrial cancer surgery. Gynecol. Oncol..

[26] Tyler N., Hodkinson A., Planner C., Angelakis I., Keyworth C., Hall A., Jones P.P., Wright O.G., Keers R., Blakeman T. (2023). Transitional Care Interventions From Hospital to Community to Reduce Health Care Use and Improve Patient Outcomes: A Systematic Review and Network Meta-Analysis. JAMA Netw. Open.

[27] Binuya M.A.E., Engelhardt E.G., Schats W., Schmidt M.K., Steyerberg E.W. (2022). Methodological guidance for the evaluation and updating of clinical prediction models: A systematic review. BMC Med. Res. Methodol..

[28] Rountree L., Lin Y.-T., Liu C., Salvatore M., Admon A., Nallamothu B., Singh K., Basu A., Bu F., Mukherjee B. (2025). Reporting of Fairness Metrics in Clinical Risk Prediction Models Used for Precision Health: Scoping Review. Online J. Public Health Inform..

[29] Saleh S.N., Makam A.N., Halm E.A., Nguyen O.K. (2020). Can we predict early 7-day readmissions using a standard 30-day hospital readmission risk prediction model?. BMC Med. Inform. Decis. Mak..

[30] Meurs E.A.I.M., Siegert C.E.H., Uitvlugt E., El M.N., Stoffels R.J., Schölvinck D.W., Taverne L.F., Hulshof P.B.J.E., Ten Horn H.J.S., Noordman P.C.W. (2021). Clinical characteristics and risk factors of preventable hospital readmissions within 30 days. Sci. Rep..

