# Supplementary material for: Development and Temporal Validation of a Discharge-Based Administrative Data Risk Model for 30-Day Unplanned Readmission After Endometrial Cancer Staging Surgery
Source: Healthcare (Basel). 2026 Jul 23;14(15):2249. doi: 10.3390/healthcare14152249 (PMC13465515; doi:10.3390/healthcare14152249)
Supplement: Supplementary file 1 [file healthcare-14-02249-s001.zip › healthcare-4395710-supplementary.pdf]

File S1:TRIPOD Checklist: Prediction Model Development and Validation

Section/Topic	Item		Checklist Item	Page
**Title and abstract**				
Title	1	D;V	Identify the study as developing and/or validating a multivariable prediction model, the target population, and the outcome to be predicted.	1
Abstract	2	D;V	Provide a summary of objectives, study design, setting, participants, sample size, predictors, outcome, statistical analysis, results, and conclusions.	1-2
**Introduction**				
Background and objectives	3a	D;V	Explain the medical context (including whether diagnostic or prognostic) and rationale for developing or validating the multivariable prediction model, including references to existing models.	3
	3b	D;V	Specify the objectives, including whether the study describes the development or validation of the model or both.	3-4
**Methods**				
Source of data	4a	D;V	Describe the study design or source of data (e.g., randomized trial, cohort, or registry data), separately for the development and validation data sets, if applicable.	4-5
	4b	D;V	Specify the key study dates, including start of accrual; end of accrual; and, if applicable, end of follow-up.	4-6
Participants	5a	D;V	Specify key elements of the study setting (e.g., primary care, secondary care, general population) including number and location of centres.	4-6
	5b	D;V	Describe eligibility criteria for participants.	6
	5c	D;V	Give details of treatments received, if relevant.	NA
Outcome	6a	D;V	Clearly define the outcome that is predicted by the prediction model, including how and when assessed.	6-8
	6b	D;V	Report any actions to blind assessment of the outcome to be predicted.	NA
Predictors	7a	D;V	Clearly define all predictors used in developing or validating the multivariable prediction model, including how and when they were measured.	7
	7b	D;V	Report any actions to blind assessment of predictors for the outcome and other predictors.	7
Sample size	8	D;V	Explain how the study size was arrived at.	6-8
Missing data	9	D;V	Describe how missing data were handled (e.g., complete-case analysis, single imputation, multiple imputation) with details of any imputation method.	9
Statistical analysis methods	10a	D	Describe how predictors were handled in the analyses.	9-10
	10b	D	Specify type of model, all model-building procedures (including any predictor selection), and method for internal validation.	9-10
	10c	V	For validation, describe how the predictions were calculated.	9-10
	10d	D;V	Specify all measures used to assess model performance and, if relevant, to compare multiple models.	9-10
	10e	V	Describe any model updating (e.g., recalibration) arising from the validation, if done.	9-10
Risk groups	11	D;V	Provide details on how risk groups were created, if done.	9-10
Development vs. validation	12	V	For validation, identify any differences from the development data in setting, eligibility criteria, outcome, and predictors.	9-10
**Results**				
Participants	13a	D;V	Describe the flow of participants through the study, including the number of participants with and without the outcome and, if applicable, a summary of the follow-up time. A diagram may be helpful.	10-11
	13b	D;V	Describe the characteristics of the participants (basic demographics, clinical features, available predictors), including the number of participants with missing data for predictors and outcome.	10-14
	13c	V	For validation, show a comparison with the development data of the distribution of important variables (demographics, predictors and outcome).	
Model development	14a	D	Specify the number of participants and outcome events in each analysis.	14-17
	14b	D	If done, report the unadjusted association between each candidate predictor and outcome.	14-17
Model specification	15a	D	Present the full prediction model to allow predictions for individuals (i.e., all regression coefficients, and model intercept or baseline survival at a given time point).	14-17
	15b	D	Explain how to the use the prediction model.	14-17
Model performance	16	D;V	Report performance measures (with CIs) for the prediction model.	16-18
Model-updating	17	V	If done, report the results from any model updating (i.e., model specification, model performance).	NA
**Discussion**				
Limitations	18	D;V	Discuss any limitations of the study (such as nonrepresentative sample, few events per predictor, missing data).	22-23
Interpretation	19a	V	For validation, discuss the results with reference to performance in the development data, and any other validation data.	19-22
	19b	D;V	Give an overall interpretation of the results, considering objectives, limitations, results from similar studies, and other relevant evidence.	19-22
Implications	20	D;V	Discuss the potential clinical use of the model and implications for future research.	
**Other information**				
Supplementary information	21	D;V	Provide information about the availability of supplementary resources, such as study protocol, Web calculator, and data sets.	NA
Funding	22	D;V	Give the source of funding and the role of the funders for the present study.	NA

*Items relevant only to the development of a prediction model are denoted by D, items relating solely to a validation of a prediction model are denoted by V, and items relating to both are denoted D;V. We recommend using the TRIPOD Checklist in conjunction with the TRIPOD Explanation and Elaboration document.

Supplementary Table S1. ICD-10-PCS Codes Used to Identify Hysterectomy and Lymph Node Procedures

Proceduer Category	ICD-10-PCS Code	Official Descriptor	Procedure Type	Body Region	Approach
**Hysterectomy**	0UT90ZZ	Resection of Uterus	Total hysterectomy	Uterus	Open
	0UT94ZZ	Resection of Uterus	Total hysterectomy	Uterus	Percutaneous endoscopic
	0UT97ZZ	Resection of Uterus	Total hysterectomy	Uterus	Via natural/artificial opening
	0UT98ZZ	Resection of Uterus	Total hysterectomy	Uterus	Natural/artificial opening endoscopic
	0UTC0ZZ	Resection of Uterus and Cervix	Total hysterectomy	Uterus & cervix	Open
	0UTC4ZZ	Resection of Uterus and Cervix	Total hysterectomy	Uterus & cervix	Percutaneous endoscopic
	0UTC7ZZ	Resection of Uterus and Cervix	Total hysterectomy	Uterus & cervix	Via natural/artificial opening
	0UTC8ZZ	Resection of Uterus and Cervix	Total hysterectomy	Uterus & cervix	Natural/artificial opening endoscopic
	0UTB0ZZ	Excision of Uterus (Supracervical)	Supracervical hysterectomy	Uterus	Open
	0UTB4ZZ	Excision of Uterus (Supracervical)	Supracervical hysterectomy	Uterus	Percutaneous endoscopic
	0UTB7ZZ	Excision of Uterus (Supracervical)	Supracervical hysterectomy	Uterus	Via natural/artificial opening
	0UTB8ZZ	Excision of Uterus (Supracervical)	Supracervical hysterectomy	Uterus	Natural/artificial opening endoscopic
**Lymph node procedures**	07BC0ZZ	Excision of Pelvis Lymphatic	Lymph node biopsy/excision	Pelvic	Open
	07BC0ZX	Excision of Pelvis Lymphatic (Diagnostic)	Lymph node biopsy	Pelvic	Open
	07BC3ZZ	Excision of Pelvis Lymphatic	Lymph node biopsy/excision	Pelvic	Percutaneous
	07BC3ZX	Excision of Pelvis Lymphatic (Diagnostic)	Lymph node biopsy	Pelvic	Percutaneous
	07BC4ZZ	Excision of Pelvis Lymphatic	Lymph node biopsy/excision	Pelvic	Percutaneous endoscopic
	07BC4ZX	Excision of Pelvis Lymphatic (Diagnostic)	Lymph node biopsy	Pelvic	Percutaneous endoscopic
	07BD0ZZ	Excision of Aortic Lymphatic	Lymph node biopsy/excision	Para-aortic	Open
	07BD0ZX	Excision of Aortic	Lymph node biopsy	Para-aortic	Open

	Lymphatic (Diagnostic)			
07BD3ZZ	Excision of Aortic Lymphatic	Lymph node biopsy/excision	Para-aortic	Percutaneous
07BD3ZX	Excision of Aortic Lymphatic (Diagnostic)	Lymph node biopsy	Para-aortic	Percutaneous
07BD4ZZ	Excision of Aortic Lymphatic	Lymph node biopsy/excision	Para-aortic	Percutaneous endoscopic
07BD4ZX	Excision of Aortic Lymphatic (Diagnostic)	Lymph node biopsy	Para-aortic	Percutaneous endoscopic
07TC0ZZ	Resection of Pelvis Lymphatic	Lymphadenectomy	Pelvic	Open
07TC4ZZ	Resection of Pelvis Lymphatic	Lymphadenectomy	Pelvic	Percutaneous endoscopic
07TD0ZZ	Resection of Aortic Lymphatic	Lymphadenectomy	Para-aortic	Open
07TD4ZZ	Resection of Aortic Lymphatic	Lymphadenectomy	Para-aortic	Percutaneous endoscopic

Abbreviation: ICD-10-PCS, International Classification of Diseases, Tenth Revision, Procedure Coding System.

Supplementary Table S2. Study Variables Derived from the Nationwide Readmissions Database (NRD)

Study Variable	NRD Field / Source	Domain	Type	Coding / Levels (Study Use)	Model Transformation / Reference	Missing (%)	Missing Handling	Notes
Age (years)	AGE (Core file)	Demographics	Continuous	Years at admission	Restricted cubic spline (3 df), reference = median	0.08	Complete-case	Standard risk factor
Female	FEMALE (Core file)	Demographics	Binary	1=Female, 0=Male	Not included in model (female-only cohort QC variable)	0	Not applicable	QC variable
Primary expected payer	PAY1	Socioeconomic	Categorical	Medicare; Medicaid; Private/HMO; Self-pay; Other	Reference = Private/HMO	0.72	Complete-case	Access to care proxy
ZIP income quartile	ZIPINC_QRTL	Socioeconomic	Ordinal	Q1–Q4	Reference = Q4	8.01	Multiple imputation (m=10)	Area-level SES proxy
ED utilization	HCUP_ED	Admission/Encounter	Binary	1=ED, 0=No ED	Reference = 0	0.24	Complete-case	Severity proxy
Weekend admission	AWEEKEND	Admission/Encounter	Binary	1=Weekend, 0=Weekday	Reference = 0	0.1	Complete-case	Care access proxy
Admission type	ATYPE / ELECTIVE	Admission/Encounter	Binary	Elective vs Non-elective	Reference = Elective	0.37	Complete-case	Acute presentation
Length of stay (days)	LOS	In-hospital course	Continuous	Days	Restricted cubic spline (3 df), reference = median	0.13	Complete-case	Severity/recovery proxy
Major OR procedure	PCLASS_ORPROC	In-hospital course	Binary	1=Yes, 0=No	Reference = 0	0.72	Complete-case	Surgical complexity
Discharge disposition	DISPUNIFORM	Disposition	Categorical	Home, home health, facility, etc.	Reference = Home/self-care	0.56	Complete-case	Functional status proxy
Hospital region	HOSP_REGION	Hospital-level	Categorical	Northeast,	Reference =	0.09	Complete-	Geographic variation

				Midwest, South, West	Northeast		case	
Teaching status	HOSP_LOCTEACH	Hospital-level	Binary	Teaching vs Non-teaching	Reference = Non-teaching	0.23	Complete-case	Academic center proxy
Bed size	HOSP_BEDSIZE	Hospital-level	Categorical	Small, Medium, Large	Reference = Small	0.33	Complete-case	Capacity proxy
Ownership/control	HOSP_CONTROL	Hospital-level	Categorical	Gov, nonprofit, investor-owned	Reference = Nonprofit	0.48	Complete-case	System-level differences
Elixhauser readmission risk index	AHRQ Elixhauser (ICD-10-CM v2022.1)	Comorbidity	Continuous	Composite score	Per 1-point increase	0.66	Complete-case	Comorbidity burden
Elixhauser mortality risk index	AHRQ Elixhauser (ICD-10-CM v2022.1)	Comorbidity	Continuous	Composite score	Per 1-point increase	0.67	Complete-case	Baseline risk adjustment

Abbreviation: NRD, Nationwide Readmissions Database; ED, emergency department; SES, socioeconomic status; OR, operating room; AHRQ, Agency for Healthcare Research and Quality.
